# The Relationship between Sleep, Chronotype, and Dental Caries—A Narrative Review

**DOI:** 10.3390/clockssleep5020023

**Published:** 2023-05-15

**Authors:** Anamaria Kurtović, Jasminka Talapko, Sanja Bekić, Ivana Škrlec

**Affiliations:** 1 Faculty of Dental Medicine and Health, Josip Juraj Strossmayer University of Osijek, 31000 Osijek, Croatia; 2 Faculty of Medicine, Josip Juraj Strossmayer University of Osijek, 31000 Osijek, Croatia; 3 Family Medicine Practice, 31000 Osijek, Croatia

**Keywords:** circadian rhythm, chronotype, dental caries, oral cavity, sleep, sleeping habits

## Abstract

This article provides an overview of how sleep and circadian rhythm disturbances mutually influence the occurrence of dental caries and how it is possible to reduce the risk of circadian rhythm disturbances, sleep, and associated adverse effects. Dental caries is a global problem worldwide that contributes to sociological limitations. Numerous factors influence the occurrence of dental caries, from socioeconomic factors to cariogenic bacteria, dietary habits, and oral hygiene. However, sleep disorders and circadian rhythm disturbances represent a new approach in the fight against the increasing prevalence of dental caries worldwide. Bacteria in the oral cavity and the oral microbiome are mainly responsible for the development of caries, and saliva plays an important role in their regulation. The circadian rhythm regulates numerous physiological functions, including sleep and saliva production. Disturbances in sleep and circadian rhythms affect saliva production, which impacts the development of dental caries, as saliva is necessary for regulating and maintaining oral health, especially for controlling oral infections. A person’s preference for a particular time of day depends on the circadian rhythm called chronotype. Individuals with an evening chronotype have a less healthy lifestyle that can lead to a higher caries risk than individuals with a morning chronotype. Because circadian rhythms are critical to maintaining sleep homeostasis and oral health, sleep disturbances can disrupt circadian rhythms and lead to a vicious cycle.

## 1. Introduction

Dental caries, also known as tooth decay, is a prevalent noncommunicable disease affecting approximately 2.3 billion people worldwide, which accounts for 32% of the global population, as stated by the World Health Organization (WHO) [1]. The prevalence of dental caries varies by region and country, with the highest rates in low- and middle-income countries, where it commonly affects children as young as three years old [2,3]. The disease is caused by bacteria in the mouth, which produce acid when exposed to sugars and carbohydrates in the food, leading to tooth decay over time [4,5].

Sleep is critical for maintaining normal brain function and controlling the functions of many other body systems. In contrast, insufficient sleep is associated with decreased immunity, increased inflammation, and susceptibility to bacterial infections [6,7,8]. Therefore, poor sleep habits are a risk factor for dental caries, as short sleep duration increases susceptibility to the cariogenic bacteria responsible for tooth decay [9,10]. Lack of sleep also leads to an increased appetite, promoting the development of caries when sugary foods are consumed [11]. In addition, sleep-related breathing disorders, such as mouth breathing, can cause oral changes and contribute to dental issues [12].

The circadian rhythm helps regulate many physiological functions, including sleep–wake cycles, hormone production, body temperature, and metabolism [13]. Understanding the circadian rhythm and its effects on our health is important for developing strategies to promote health and prevent disease [14]. For example, research has shown that maintaining a regular sleep–wake rhythm and avoiding circadian rhythm disruptions can help reduce the risk of several chronic health conditions, including dental caries. The rhythm of human preference for timing sleep and wakefulness in relation to the 24 h solar day determines whether a person is a morning or evening person—or their chronotype [15]. Studies have shown that people with an evening chronotype are at higher risk for certain health conditions than people with a morning chronotype [16]. Some of these include mood disorders, personality disorders, anxiety disorders, substance use disorders, sleep apnea, insomnia, asthma, arterial hypertension, type 2 diabetes, and infertility, as reported by Partonen et al. [16]. In addition, individuals with an evening chronotype are more likely to have unhealthy lifestyle habits, such as a diet high in fat and sugar and less physical activity [17,18]. These behaviors may increase the risk of dental caries. On the other hand, individuals with morning preferences are more likely to exhibit healthy habits and fewer diseases [19]. In the present study, scoping and systematic reviews were not performed because a search of the main databases, such as WoS, Medline, Scopus, and RCA, did not find any studies that simultaneously investigated the association between sleep and chronotype and caries’ development. In fact, the studies investigated either the association between sleep and caries or between chronotype and caries, but not both. Thus, a narrative approach was adopted for this topic, with certain limitations, such as the lack of a well-defined database search protocol and no quality assessment of the included studies, although narrative reviews can be comprehensive. Therefore, the aim of this narrative review is to provide an overview of the current knowledge on the relationship between circadian rhythm, sleep, and the prevalence of dental caries.

## 2. Dental Caries

The prevalence of dental caries is influenced by various factors, including diet, oral hygiene practices, access to oral health care, and socioeconomic status [20,21]. Dental caries affect 60% to 90% of school-aged children [22]. The WHO recommends implementing community-based programs to prevent dental caries and promote oral health, such as fluoridation of water supplies and sugar reduction campaigns [23].

The oral microbiota plays a crucial role in dental health and demineralization. The oral microbiota helps maintain the health of the teeth and gums by creating a protective biofilm on the tooth surfaces, which helps prevent the colonization of harmful bacteria. However, if the oral microbiota is disrupted or imbalanced, harmful bacteria can proliferate and cause dental problems such as tooth decay and gum disease [24]. Several bacteria species are associated with dental caries. The most significant cariogenic bacteria include *Streptococcus mutans*, *Streptococcus sobrinus*, *Lactobacillus acidophilus*, and *Actinomyces* species [25]. These bacteria species produce acids that dissolve the minerals in the tooth’s enamel, leading to demineralization and, ultimately, cavities [26]. After consuming sugary or starchy foods, bacteria ferment the carbohydrates, thereby producing lactic acid, which lowers the pH in the oral cavity and contributes to tooth decay [27]. This acidic environment can weaken the tooth’s enamel by demineralization and allow the bacteria to penetrate the tooth, leading to dental caries [28]. *S. mutans* and *S. sobrinus* are considered the most important cariogenic bacteria due to their ability to produce large amounts of acid and form plaque on the tooth surface [29]. *L. acidophilus* and *Actinomyces* species are also cariogenic bacteria that can contribute to the development of dental caries [5,25]. *L. acidophilus* produces lactic acid from carbohydrates and can thrive in acidic environments [30]. *Actinomyces* species are known to produce enzymes that can break down proteins, which can contribute to the formation of cavities [31]. Preventing dental caries involves reducing the number of cariogenic bacteria in the oral cavity through good oral hygiene practices and a healthy diet [32,33]. Consuming probiotics can help promote healthy oral microbiota and prevent the proliferation of harmful bacteria [24].

Caries’ development is associated, among those mentioned, with biological, physical, environmental, and behavioral factors, such as poor saliva flow, insufficient exposure to fluoride, and low socioeconomic status [19,34]. Risk factors for the development of caries are sociodemographic, factors related to diet, factors related to oral hygiene habits, factors related to oral bacterial flora, and other factors such as genetic background and smoking [21]. Thus, poor oral hygiene increases dental caries risk by not regularly removing plaque from the teeth through brushing and flossing [35]. Consuming foods and drinks high in sugar or acid can contribute to tooth decay [23]. Moreover, a diet lacking essential nutrients such as calcium, vitamin D, and phosphorus can weaken tooth enamel and increase the risk of dental caries [36]. A lack of saliva in the mouth can increase the risk of dental caries because saliva helps to neutralize acid and wash away food particles and bacteria. Certain medications, medical conditions, and lifestyle factors such as smoking can cause dry mouth or xerostomia [37]. Fluoride is a mineral that helps strengthen tooth enamel and protect against tooth decay. Insufficient fluoride exposure, either through drinking water, toothpaste, or other sources, can increase the risk of dental caries [38]. Children and older adults are at higher risk of dental caries. Children’s teeth are still developing and may not fully form enamel, while older adults may have more exposed root surfaces due to gum recession [39,40]. Some individuals may be more genetically predisposed to developing dental caries due to differences in the structure and composition of their teeth [41]. The most common factors contributing to caries’ development are presented in Table 1. Parents’ habits can influence the outcome of dental caries in children. This may be because children often share their parents’ eating and drinking habits and may also learn oral hygiene practices from their parents [42,43]. Moreover, parental smoking habits are associated with dental caries in children, and children exposed to secondhand smoke have higher rates of caries than other children [44,45].

The commonly used method for measuring dental caries’ occurrence in populations is the DMFT index. DMFT stands for decayed, missing, and filled teeth. The index is calculated by counting the number of teeth in a person’s mouth that have decayed, are missing due to caries, or have been filled due to caries [47]. A higher DMFT score indicates more significant caries in the individual [48]. The DMFT index can be used to assess the prevalence and severity of dental caries in a population, as well as to monitor changes in caries’ occurrence over time. It is also helpful for evaluating the effectiveness of oral health programs and interventions to reduce caries’ prevalence [49].

### Saliva

In addition to the DMFT index, caries biomarkers are reduced saliva flow and an increased number of *S. mutans*. A decreased quantity or quality of saliva can increase the risk of dental caries [50]. Saliva is essential for regulating the oral microbiome and maintaining oral health, especially in preventing oral diseases and controlling oral infections [51]. Normal saliva secretion and saliva flow are directly related to oral health. Decreased saliva flow is an important indicator of oral health, and disorders of the salivary glands are the main factor affecting the decrease in saliva flow [51]. Saliva has the most significant influence on caries’ progression [52]. A reduction in saliva at night favors the progression of caries [52]. Moreover, saliva’s buffering capacity depends on saliva’s production at rest [53], and it is considered that the risk of caries increases in children who sleep less due to a decrease in saliva production [53]. Interestingly, the higher the DMFT index, the lower the saliva flow [54].

**Figure 1:**
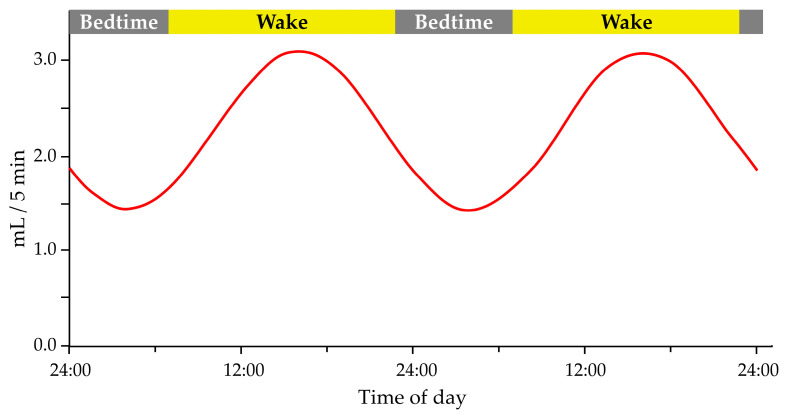
The circadian pattern of salivary flow rate. Modified according to Dawes 1972 [55]. Adapted with permission form Ref. [55]. 2023, John Wiley and Sons.

Saliva flow and production have been shown to exhibit a circadian pattern (Figure 1), with peak flow rates occurring during the day and lower flow rates at night [55,56]. The suprachiasmatic nucleus regulates the circadian rhythm of salivary flow and production, the body’s central circadian clock in the hypothalamus [55]. Saliva flow, salivary protein concentration, and electrolytes have a circadian pattern and are essential in maintaining and protecting oral health [52]. Saliva flow is weak in the morning, increases in the afternoon, and then decreases [57]. The circadian rhythm of the salivary glands plays a vital role in controlling food intake and the immune system because it affects the flow of saliva and the ionic composition [51,58]. Changes in saliva flow and composition can affect oral health, as saliva helps to neutralize acids produced by oral bacteria, remineralize tooth enamel, and lubricate the mouth [59]. Saliva is important for maintaining tooth remineralization, and people with xerostomia have an increased number of *S. mutans* and other acidogenic species, which favors caries’ development [52]. In addition, sleep disturbances can lead to circadian rhythm disturbances, affecting saliva production, which partially explains the connection between insufficient sleep and caries’ development [50,60]. Furthermore, disruption of the circadian rhythm, such as chronic sleep disturbance, may affect saliva flow and composition, contributing to oral health problems, including dental caries [54].

## 3. Sleep Homeostasis

Sleep is an essentially biological process visible in many organisms [60]. Lack of sleep is a global phenomenon, and epidemiological data show that insufficient sleep negatively affects human physical health [10], including the production and flow of saliva. The underlying mechanisms of sleep include the modulation of inflammatory immune mechanisms. Sleep disorders can be categorized as insomnia, sleep-related breathing disorders, and sleep–wake circadian rhythm disorders [61]. Sleep problems in childhood can affect the child’s health and development [62]. For example, inadequate sleep is associated with early childhood dental caries [63], and children’s dental problems affect their sleep [64]. On the other hand, healthy sleeping habits are associated with strengthening the immune system and reducing the risk of heart and metabolic disorders [26].

### 3.1. Insufficient Sleep and Caries

There is a connection between the amount of sleep and dental caries. Studies have shown that poor sleep or poor sleep quality can increase the risk of developing dental caries [27,48]. Moreover, some studies have suggested that poor sleep quality and insufficient sleep may be associated with a higher risk of dental caries, as reflected by the DMFT index [26,63,64]. One reason for this connection is that insufficient sleep can lead to changes in the body’s immune system, making it less effective at fighting off bacteria that cause dental caries [65]. Additionally, during sleep, saliva production is reduced, which can cause a decrease in the neutralization of acids produced by oral bacteria that can lead to enamel erosion and tooth decay [66]. Therefore, poor sleep quality and insufficient sleep may lead to changes in saliva production and composition, which could contribute to the development of dental caries [67]. Sleep habits contributing to caries’ occurrence are given in Table 2.

Insufficient sleep has been associated with an increased risk of dental caries in adolescents [64]. Several studies have investigated the relationship between sleep duration or sleep quality and dental caries in adolescents, with some suggesting that insufficient sleep may be a risk factor for caries [26,63,64,65]. Thus, Sardana et al. reported that irregular or late bedtimes and fewer hours of sleep were independent risk factors for caries in early childhood [26]. Ogawa et al. reached a similar conclusion in their study, reporting that sleep duration was independently associated with caries in early childhood [68]. Duration and quality of sleep were significantly related to symptoms of dental disease in a study by Choi et al. [65]. Moro et al. took a slightly different approach and examined sleep problems in school children due to dental issues and came to similar conclusions—sleep problems in school children are associated with untreated dental caries [64]. Although these studies were conducted on different continents, they all came to one solid conclusion—sleep is a risk factor for the development of dental caries. The mechanisms by which insufficient sleep may contribute to adolescents’ caries’ development are not fully understood. Still, it has been suggested that changes in saliva flow and composition, as well as alterations in dietary habits and oral hygiene practices, may play a role [2,5,39]. Insufficient sleep can affect the production and composition of saliva, reducing its ability to neutralize acids produced by oral bacteria. In addition, insufficient sleep has been linked to poor dietary habits, such as increased consumption of sugary and acidic foods and drinks, which can contribute to the development of dental caries [39].

Having enough quality sleep is vital for overall health, including dental health. It can help maintain a healthy immune system and adequate saliva production, reducing the risk of developing dental caries [69]. Lack of sleep increases salivary glucose levels, decreases salivary flow, and increases the number of *S. mutans*, which can alter the levels of inflammatory cytokines and modify the caries formation process [11,26,68].

Moreover, children’s bedtime can have an impact on caries’ prevalence. Studies have shown that individuals who go to bed later at night risk developing dental caries more than those who go earlier [70]. In addition, later going to bed [9,61] and short night sleep duration are associated with an increased risk of caries [50]. In children, the sleeping routine is also essential, and those children who had an irregular bedtime had a higher prevalence of caries [50]. One possible explanation for this association is that staying up late at night can lead to irregular sleep patterns, disrupting the circadian rhythm and decreasing saliva production, making it harder for the body to neutralize the acids produced by oral bacteria [71].

Additionally, individuals who stay up late may be more likely to consume sugary or acidic foods and drinks, which can also contribute to tooth decay [9]. In addition, due to going to sleep later, hormones related to appetite are stimulated, which leads to increased food intake and snacking—night eating [9,27]. Furthermore, staying up late can lead to poor oral hygiene practices, such as skipping brushing or flossing before bed [48]. Neglecting these practices can allow oral bacteria to thrive, increasing the risk of developing dental caries. Children who stay awake longer have a higher risk of developing caries and brush their teeth less often. Shorter sleep duration is associated with increased *S. mutans* in saliva [9,27]. Thus, the research showed that children who went to sleep irregularly had an increased risk of caries’ development [26]. Children who went to sleep after 11 p.m. also had an increased risk of tooth decay [26]. The prevalence of caries was lower in children who went to bed before 9 p.m. [70].

Sleep habits can have a significant impact on a child’s oral health. Poor sleep habits, such as fragmented sleep [61] and unhealthy sleep habits of parents, have been associated with a higher prevalence of dental caries in children [63]. Allowing children to eat sugary products before bedtime has also been associated with higher caries’ prevalence [48]. In contrast, maintaining a healthy bedtime routine may lead to healthier teeth in children [48]. In addition, it is important to note that dental caries can negatively affect family life and lead to sleep disturbances in children [22]. Therefore, establishing a healthy lifestyle is important for preventing dental caries in children [63], and children who slept longer had a lower prevalence of caries [71]. Maintaining a regular bedtime and good sleep hygiene practices can help reduce the risk of developing dental caries [48]. Additionally, practicing good oral hygiene habits and a healthy diet can help promote oral health and reduce the risk of dental caries [72].

### 3.2. Sleep Duration and Caries Prevalence

Sleep duration has been linked to caries’ development, with short and long sleep durations associated with an increased risk of dental caries. Short sleep duration, typically defined as less than 7 h per night, has been associated with an increased risk of dental caries in children and adults [26,63]. Long sleep duration, typically defined as more than 9 h per night, has also been associated with an increased risk of dental caries, particularly in children [64]. Research has shown that children who slept less than 8 h during the night had an increased risk of caries compared to children who slept more than 11 h [26,53]. Insufficient sleep in children is associated with an increased risk of tooth decay. The shorter the sleep duration, the greater the risk [50,53]. A possible explanation is that with fewer hours of sleep, there is an increase in the sympathetic nervous system, which causes a weakening of the immune system. Since caries is a bacterial infection, the risk of developing caries increases with a decrease in immune defense against pathogens [53]. Untreated caries can cause toothache, abscess, and cellulitis [26], contributing to sleep disorders in children [26]. Table 3 summarizes the results of the review studies on the association between sleep habits and sleep duration and the incidence of dental caries in children.

The exact mechanisms by which sleep duration influences caries’ development are poorly understood. Still, changes in saliva flow, oral microbiota composition, and dietary habits may play a role in caries’ development. For example, short sleep duration has been associated with decreased saliva flow, which can reduce the ability of saliva to neutralize acids produced by oral bacteria and remineralize tooth enamel [59,73]. Long sleep duration may also disrupt normal saliva flow patterns, leading to changes in oral microbiota composition and an increased risk of caries [74]. In addition, short and long sleep durations have been linked to poor dietary habits, including increased consumption of sugary and acidic foods and drinks, which can contribute to developing dental caries [26]. Maintaining a regular sleep schedule and having adequate sleep each night may be necessary for promoting good oral health and reducing the risk of dental caries [75]. This includes establishing a regular sleep schedule, limiting screen time before bedtime, and creating a comfortable sleep environment.

**Table 3:** Characteristics of studies comparing the association between sleeping habits and the dental caries’ incidence.

Study	Country	Study Design	Population Description	Sleep Assessment	Study Period	Age Range	Sample Size (Sex)	Primary Outcome
Watanabe et al., 2014 [69]	Japan	Cohort study	Children participated in routine dental examinations at 1.5 years old at Kobe City Public Health Center in Japan	Parents or guardians reported children’s bedtime (before 9 p.m., between 9 and 11 p.m., after 11 p.m., or irregular)	Between June 2006 and August 2009 and between April 2008 and March 2011	1.5 years old at baseline and 3 years at a dental check-up	31,202 children (16,052 male and 15,150 female)	Late bedtime is a risk factor for the development of dental caries
Chen et al., 2018 [50]	Japan	Cohort study	Municipal health check-ups for children aged 0–3 years in Kobe City, Japan	Parents reported their child’s wake time and bedtime on a standardized questionnaire	From 31 March 2004 to 1 April 2014	1.5 years old at baseline and 3 at a dental check-up	71,069 children (36,245 male and 34,824 female)	Late bedtime and short sleep duration are associated with an increased risk of caries
Kitsaras et al., 2018 [48]	UK	Cross-sectional study	Participants were recruited through an active study on General Dental Anaesthetic teeth extraction and through General Dental Practices	Interactive text survey for the assessment of bedtime routines	From March to June 2017	Range from 3 to 5 years old	50 children (24 male and 26 female)	Children with optimal bedtime routines had fewer cavities and missing teeth than children with suboptimal bedtime routines
Zhou et al., 2019 [71]	China	Cross-sectional study	Children from 3 to 5 years old residing in Zhejiang province, living there for more than six months	Structured questionnaire completed by the children’s parents or guardian	From January to June 2016	Range from 3 to 5 years	1591 children (821 male and 770 female)	Longer sleeping (≥12 h) is associated with lower caries’ prevalence
Asaka et al., 2020 [53]	Japan	Cross-sectional study	Children participated in the Super Shokuiku School Project in Takaoka City, Toyama Prefecture	Parents completed the questionnaires on their child’s sleep duration (short sleep duration was defined as <8 h)	March 2016	Range from 6 to 12 years	1699 children (848 male and 851 female)	Short sleep duration is associated with higher dental caries’ prevalence
Alqaderi et al., 2020 [9]	Kuwait	Cohort study	Children across the six governorates of Kuwait	Questionnaire-based interviews with parents or guardians on child’s bedtime and sleep duration	In 2012 and a dental check-up in 2014	10 years at baseline and 12 years at a check-up	5456 children (2103 male and 3353 female)	Late bedtime is associated with increased dental caries’ incidence
Ogawa et al., 2022 [63]	Japan	Observational cross-sectional study	Children in five kindergartens, nursery schools, and early childhood education and care centers in Chitose, Japan	Parents filled out a questionnaire on their child’s sleeping status	During October 2020	Range from 3 to 6 years	332 children (178 male and 154 female)	The negative correlation between sleep durations and the number of caries
Topaloglu-Ak et al., 2022 [61]	Turkey	Cross-sectional study	Children referred to the Department of Pediatric Dentistry at the Faculty of Dentistry of Istanbul Aydin University	Children’s Sleep Habits Questionnaire completed by child’s parents	From 2 March 2020 to 29 May 2020	Range from 6 to 13 years	100 children (45 male and 55 female)	The presence of caries is associated with awakening from sleep at night and sleep fragmentation
Arroyo Buenestado et al., 2023 [76]	Spain	Cross-sectional study	Dental clinic Clínica María Isabel Rodríguez, Pozoblanco, Córdoba	Parents or caregiver filled out a Spanish version of the Paediatric Sleep Questionnaire	From January to March 2021	Range from 2 to 5 years	80 children (40 male and 40 female)	Higher caries’ prevalence among later bedtime and later wake-up time

## 4. Circadian Rhythm

Circadian rhythm is the 24 h physiological and behavioral cycle in living organisms, including humans [77], and is vital in sleep–wake cycles. An internal biological clock regulates these processes in the brain’s suprachiasmatic nucleus, responding to external cues such as light and dark [51], and plays an essential role in sleep regulation. Circadian rhythm is important to humans for several reasons. First, it helps to regulate the sleep–wake cycle, which is critical for maintaining physical and mental health. Second, disruptions to the circadian rhythm can lead to sleep disturbances and other health problems [77]. In addition to sleep, the circadian rhythm also helps to regulate many other physiological processes, including digestion, hormone production, and immune function. Third, disruptions to the circadian rhythm can lead to various health problems, including metabolic disorders, cardiovascular disease, and certain types of cancer [78,79]. Finally, disturbance of the rhythm and dysfunction of the circadian clock can result in various oral pathological conditions [51].

The circadian rhythm affects melatonin secretion [80], and the reduced secretion of melatonin leads to a decrease in antioxidants, which results in higher production of reactive oxygen species in saliva [54]. By decreasing antioxidants, including melatonin, the increased level of reactive oxygen species in saliva leads to changes in salivary oxidative biochemistry, which has been associated with the development of dental caries [81]. Since melatonin is a circadian hormone, another link between circadian rhythm and the occurrence of dental caries is evident from the above. In addition, there is a decrease in the pH of saliva, which causes an acidic condition in the oral cavity and favors the development of acidic and acidogenic bacteria such as *S. mutans*. Additionally, *S. mutans* creates biofilm and dental plaque, producing the organic acids that diffuse into enamel and dentin, leading to increased demineralization and decreased remineralization of teeth [54]. Therefore, circadian rhythm disruption is a potential risk factor for increased DMFT index. Disruption of the circadian rhythm may increase the risk of dental caries, as reflected by the DMFT index [19], due to it affecting saliva production and composition, reducing immune function, altering oral microbiota composition, and influencing dietary habits, all of which can contribute to dental caries [82].

### 4.1. Chronotype and Caries’ Prevalence

Chronotype refers to the innate biological predisposition of an individual to prefer specific sleep–wake patterns [83]. There are three categories of chronotypes: morning, intermediate, and evening. Morning types prefer to wake up early and feel most alert during the early hours of the day. Evening types prefer to stay up late at night and feel most alert later in the day. Intermediate types fall somewhere between and may not strongly prefer either morning or evening [84]. Genetics largely determines chronotype and can be influenced by age, lifestyle, and environmental factors. Understanding an individual’s chronotype can help optimize their sleep–wake cycle. It can inform the best times to sleep, wake up, and engage in daily activities [15]. Individuals with an evening chronotype may be more likely to have poor sleep quality and experience sleep disorders such as insomnia and sleep apnea [85,86,87]. Poor sleep quality and sleep disorders can negatively affect overall health, including an increased risk of obesity, diabetes, cardiovascular disease, and depression [19]. Morning-type adolescents had lower DMFT scores than evening types [19]. One possible explanation for this association is that individuals with an evening chronotype may have irregular sleep patterns, leading to decreased saliva production and poor oral hygiene practices, which can increase the risk of dental caries [57]. Additionally, evening chronotypes may consume more sugary or acidic foods and drinks, which can also contribute to tooth decay [19,88].

Individuals with an evening chronotype may have a delay in the timing of saliva production compared to those with a morning chronotype [51,85]. This delay can decrease saliva production in the morning, increasing the risk of dental caries. Conversely, morning chronotype is a protective factor for caries [89]. Furthermore, studies have shown that individuals with an evening chronotype may have a higher prevalence of sleep disorders such as sleep apnea [90], which can cause dry mouth and reduce saliva production. This reduction in saliva can increase the risk of developing dental caries [85]. However, it is important to note that while chronotype can influence saliva production, many other factors can also affect it.

Moreover, oral health in people with an evening chronotype is worse due to night eating syndrome. Frequent night eating predicts poor oral health significantly [88,91]. Children with daily life habits related to the evening chronotype have a higher prevalence of dental caries [57]. Eating late at night when saliva secretion is reduced can lead to a higher prevalence of caries [57]. Some studies have shown that evening chronotypes have a higher risk of periodontal disease and caries precisely because of night eating and insomnia, which is much less represented in morning chronotypes [88]. As the evening chronotype is associated with poor habits (skipping breakfast, brushing teeth less often, eating at night), evening-type adolescents had worse oral health and a higher risk of caries than morning types [88,89]. However, the circadian rhythm changes throughout life, and morning fatigue is more common during puberty than later in life [89]. That is why parents’ healthy habits—regular bedtime, breakfast, reduced intake of sweets and juices, and oral hygiene—are important factors in preventing caries in children [68]. Individuals with evening chronotype may have shorter sleep duration, delayed sleep onset, and irregular sleep patterns [85], which could affect saliva production, reduce immune function, and alter oral microbiota composition, all of which are factors that can contribute to the development of dental caries [25,92]. Table 4 presents data from the studies included in the present review on the association between chronotype and the risk of developing dental caries assessed by the DMFT index. 

### 4.2. Importance of Circadian Rhythm and Sleep Homeostasis in Caries’ Development

**Figure 2:**
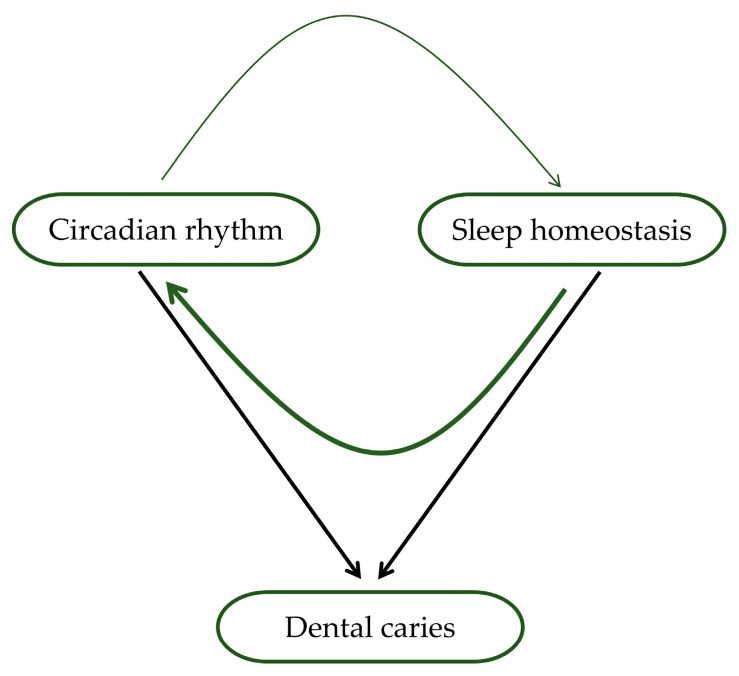
Association of circadian rhythm, sleep, and dental caries. Circadian rhythm and sleep homeostasis affect both sleep and waking, affecting the production and composition of saliva, which directly affects the development of dental caries (shown by black arrows). In addition, circadian rhythm and sleep mutually influence functioning (indicated by green arrows). The thickness of the arrows symbolizes the strength of this hypothesized effect, where the current data suggest that the impact of sleep homeostasis on circadian rhythm is more significant than the reciprocal influence.

The circadian rhythm regulates the sleep–wake cycle, and poor sleep can disrupt the circadian rhythm and vice versa [94]. These two processes determine most aspects of sleep and related variables such as sleepiness and wakefulness [95]. Disrupting the circadian rhythm can also lead to poor sleep quality, contributing to dental caries’ development [63,68]. As mentioned, the circadian rhythm affects saliva production, which is essential in maintaining oral health by neutralizing acids produced by oral bacteria that can cause tooth decay [55]. Disrupting the circadian rhythm through poor sleep can lead to inadequate saliva production, increasing the risk of developing dental caries [50]. A healthy circadian rhythm will benefit sleep, and good sleep will strengthen the creation of the rhythm (Figure 2). Contrarily, disturbed sleep can result in a less functional circadian rhythm, reducing sleep quality [95]. Conversely, dental caries can also disrupt the circadian rhythm. In addition, pain from tooth decay can interfere with sleep and lead to poor sleep quality, further disrupting the circadian rhythm [51,66]. This disruption can then cause a decrease in saliva production and immune function, making it harder for the body to fight off bacteria that cause tooth decay.

The circadian rhythm is crucial in maintaining sleep quality and oral health. Disruptions to this rhythm from poor sleep or dental caries can lead to a vicious cycle of poor sleep and oral health outcomes. Therefore, it is essential to maintain good sleep habits and seek treatment for dental caries promptly to minimize the risk of disrupting the circadian rhythm and its associated adverse effects.

## 5. Conclusions

Circadian rhythm disruption, sleep disorders, increased consumption and production of processed food rich in sugars and saturated fats, and insufficient education about oral health that is not in line with continuous economic growth lead to a high caries’ prevalence. In addition, parents’ lifestyle significantly impacts children’s habits and can lead to sleep and circadian rhythm disruptions, resulting in a higher prevalence of dental caries. Promoting healthy eating habits, good oral hygiene, adequate sleep, and managing stress all play a role in reducing the risk of caries. However, modern lifestyles and habits can increase the risk of developing caries. Therefore, acquiring healthy habits from an early age is critical to preventing or reducing the negative consequences of tooth decay in children later in life. In addition, regular dental check-ups and preventive treatments, such as the use of fluoride and dental sealants, may be necessary for maintaining good oral health. In addition, individual patient counseling should be encouraged, and caries prevention programs should consider individuals’ circadian rhythms and sleep habits.

## Figures and Tables

**Table 1:** Factors that contribute to the development of caries [21,46].

Sociodemographic Factors	Dietary Factors	Oral Hygiene
Low socioeconomic status	Daily sweet snacks	<2 daily brushings
Parental smoking	High sugar foods	Lack of fluoride toothpaste
Long screen time	Daily sweet drinks	
Gender (male)	Frequently snaking	
	Nighttime eating	
	Breakfast skipping	
	Vitamin D deficiency	
	Low levels of calcium	

**Table 2:** Sleep habits that contribute to caries’ occurrence. Adapted with permission form Ref. [11]. 2023, Elsevier.

Insufficient Sleep	Sleep Duration	Sleep Disorders
Irregular bedtime routines	Less than 8 h	Insomnia
Late bedtime (after 11 p.m.)	Longer than 10 h	Sleep-related breathing disorders
Sleep fragmentation		Sleep–wake circadian rhythm disorders

**Table 4:** Characteristics of studies comparing the association of chronotype and the risk of dental caries assessed by the DMFT index.

Study	Country	Study Design	Population Description	Chronotype Measurement	Study Period	Age Range	Sample Size Sex	Primary Outcome
Lundgren et al., 2016 [89]	Sweden	A comparative cross-sectional, case–control study	Patients at the Public Dental Service in Uppsala County Council examined during 2004 and 2005	The instrument developed by Östberg in 1973 and modified by Torvall and Akerstedt in 1980	2006	Adolescents aged 15 to 16 years	196 adolescents (101 male and 95 female)	The evening type had a higher risk of caries than the morning type
da Silveira et al., 2018 [19]	Brazil	Cross-sectional study	Adolescents aged 12 years of all public and private schools in the municipality of Brumadinho, in the state of Minas Gerais participated in the study	Brazilian Portuguese version of the Puberty and Phase Preference Scale (PPPS)	Between August and December 2016	Adolescents aged 12 years	245 adolescents (109 male and 136 female)	Chronotype was not associated with DMFT scores
Nishide et al., 2019 [57]	Japan	Cross-sectional study	Outpatients age range 1 to 16 in the university hospital in Japan at the Division of Dentistry for Children and Disabled Persons	Self-reported sleep–wake time	-	Children aged 1 to 16 years (7.2 ± 3.5 years)	140 children (77 male and 63 female)	Evening type has a higher prevalence of dental caries
Kurnaz et al., 2020 [89]	Turkey	Cross-sectional study	Patients applied at the dental clinic of the Faculty of Dentistry of Kutahya Health Sciences University	Morningness–Eveningness Questionnaire (MEQ)	Between March and September 2018	Patients aged 18 to 63 years (36.02 ± 8.91 years)	210 patients (100 male and 110 female)	Chronotype was not associated with DMFT scores
Folayan et al., 2021 [93]	Nigeria	Cross-sectional study secondary analysis	Adolescents attending private and public primary and secondary schools in Ife Central Local Government Area, Ile-Ife, Osun State, Nigeria	Horne–Östberg Morningness–Eveningness Questionnaire (MEQ)	2019	Adolescents aged 6 to 16 years	1001 children (452 male and 549 female)	Chronotype was not associated with caries’ prevalence

## Data Availability

Not applicable.
